# Does commercialization lead to more intensive management strategies? Decision-making for the utilization of non-timber forest products in a Nahua area of the Sierra Negra, Mexico

**DOI:** 10.1186/s13002-024-00701-z

**Published:** 2024-06-20

**Authors:** Myriam A. Miranda-Gamboa, Andrea Martínez-Ballesté, Martin Ricker, Alejandro Casas, José Blancas

**Affiliations:** 1 Posgrado en Ciencias Biológicas, Coordinación de Estudios de Posgrado, Instituto de Biología, Universidad Nacional Autónoma de México, Mexico City, Mexico; 2 Laboratorio de Etnobotánica Ecológica, Jardín Botánico Instituto de Biología, Universidad Nacional Autónoma de México, Mexico City, Mexico; 3 Departamento de Botánica, Instituto de Biología, Universidad Nacional Autónoma de México, Mexico City, Mexico; 4 Laboratorio de Evolución y Manejo de Recursos Genéticos, Instituto de Investigaciones en Ecosistemas y Sustentabilidad, Universidad Nacional Autónoma de México, Morelia, Michoacán Mexico; 5 Centro de Investigación en Biodiversidad y Conservación, Universidad Autónoma de Morelos, Cuernavaca, Morelos México

**Keywords:** Socio-ecological systems, Biocultural diversity, Commercial net incomes, Incipient management practices, Rural markets

## Abstract

**Background:**

The commercialization of non-timber forest products (NTFPs) provides income for rural indigenous households. The integration of NTFPs into formal markets tends to intensify management practices to ensure production and monetary benefits. However, more research is needed to understand the motivations for managing of commercialized species. We examine the influence of social, ecological, and economic factors on traditional management and how they drive the adoption of more or less intensive practices for subsistence and commercially traded NTFPs.

**Methods:**

The study was conducted in the Nahua community of Ixtacxochitla, in the Sierra Negra of central Mexico, where we conducted free lists and semi-structured interviews in 32% of the 88 households to assess socio-ecological variables related to management practices. In addition, we interviewed local traders to assess commercial variables used in a cost–benefit model to calculate the net annual income of commercialized species. Non-metric multidimensional scaling was used to analyze relationships between socio-ecological variables and management practices. We also explored the relationship between management and commercial factors using principal component analysis.

**Results:**

We recorded 64 plant and mushroom species of NTFPs used for medicinal, ornamental, ceremonial, and edible purposes, 36 of which are commercialized in the municipal market of Coyomeapan. The commercialized species generated an average annual net income of MXN 67,526 (USD 3924) per family, with five species contributing the most. Species both used for both subsistence and commercialization were managed through incipient in situ gathering, tolerance in ex situ anthropogenic areas, and intensive protection and propagation efforts in ex situ environments. Even the five species with the highest commercial returns were managed across this gradient of practices. Key factors influencing the adoption of more intensive species management practices were feasibility of management, type of species use, ecological abundance, frequency of consumption, and cultural importance.

**Conclusions:**

The intensification of NTFPs management is not solely driven by the commercial value of the products or the level of income generated. Instead, the interaction between socio-ecological and economic factors determines the extent of management practices. The main constraint to the implementation of intensive practices has been the inability to manage species outside their natural habitats, despite their cultural significance and frequent consumption. Understanding the factors involved in the harvesting of NTFPs can serve as the basis for future research aimed at analyzing the conditions for successful and sustainable NTFPs commercialization.

## Introduction

For rural indigenous households, commercial exchange and trade of non-timber forest products (NTFPs), which are biological resources obtained from wild or managed populations [1], have been a means of entering the market and generating income that can contribute significantly to household economies [2, 3], although these incomes can sometimes be modest [4]. The extent of the economic contribution of NTFPs varies depending on the management strategies used to conserve, collect, or process them and the degree of integration of products, households, and communities into the market economy. It has been suggested that the integration of NTFPs into formal markets leads to an increase in the intensity of management practices, aimed at ensuring production and product quality, as well as increasing economic income [5–7]. However, more research is needed to fully understand the motives of management of commercialized species and the social and ecological contexts in which practices are carried out and become intensified [8, 9], and importantly, to understand the economic relationship between NTFPs commercial income and other social and cultural factors with the intensification in their management strategies [10, 11]. In this context, we consider crucial to examine the processes that drive management intensification, taking into account socio-ecological [8, 12–14] as well as economic factors involved in management and commercialization [10, 15, 16].

A conceptual framework developed to explain the level of care given to NTFPs proposes a gradient of traditional management practices ranging from the simplest, such as gathering species from wild environments, through intermediate practices related to species tolerance in anthropogenic environments, to the most complex ones involving the protection, propagation, and domestication of species in both in situ and ex situ environments [17–19]. Within these management practices, those of greater intensity and complexity are directed toward species of cultural and economic values, with the aim of reducing the risk of resource depletion and increasing the availability of culturally significant species and traded resources [10, 13, 18]. In this sense, it has been proposed that commercialization, among other factors, may influence the intensity of management practices [8–10], and as these practices become more intensive for some products, they increase the ability to meet market demands, increase economic income, and ensure the persistence of the species [5, 6, 16].

In addition to cultural and commercial values, several socio-ecological factors also influence the extent of resource management [16, 20–23]. Several authors agree that the biological and ecological characteristics of a species are critical to the feasibility of manipulating it [8, 21, 24]. Insuasti et al. [9] and Blancas et al. [17] propose that low abundance of a resource in its natural habitat is an ecological factor that influences practices aimed at increasing the quantity of the resource, when that resource is culturally valued. Also, certain biological characteristics of species, such as the length of the life cycle [8, 25], and the adaptability of a species to survive in anthropogenic environments [26], are all factors that influence management decisions.

Social factors influencing management intensity have been identified in studies conducted by Casas et al. [25] and González-Insuasti et al. [9]. They show that edible plants of high cultural value are intensively managed to guarantee quality, abundance, and accessibility. In addition, ethical, aesthetic, ceremonial, and relational values have also been documented as relevant to influencing the intensity of plant management, according to Rangel-Landa et al. [14] and Farfán-Heredia et al. [27]. Governance factors also play a role in shaping management practices [16]. Belcher et al. [5] suggest that resource management is more likely to intensify in privately owned areas than in communal areas, where management decisions are made collectively. However, González-Insuasti et al. [9] found that species that are both commercially and culturally valuable, may be intensively managed, even when they are harvested on communal lands. In addition, several studies have found that the harvesting and planning of culturally and economically important products are more often regulated by social, communal agreements [14, 17, 26, 28].

Studies examining the economic dimensions of NTFPs have typically emphasized the importance of quantifying the monetary income derived from their commercialization [3, 23, 29, 30]. However, few have focused on analyzing the link between their monetary contributions and the costs associated with management practices [6, 11, 13, 22].

Ixtacxochitla is a Nahua community located in the Sierra Negra in the state of Puebla, Mexico, where families rely on a diverse range of NTFPs to meet both subsistence needs and generate economic income through trade [31]. The NTFPs available for sale are offered in the municipal market of the Coyomeapan, where traders from different locations gather [31–33]. The variety of NTFPs managed in this community is large, and there is diversification in the practices applied for these resources [31]. Some research on the socio-ecological and economic contexts in which management intensification occurs has been previously documented in the area [8, 31, 34], and these studies have provided arguments that commercial value is one of the factors driving management intensification, as suggested by Belcher et al. [5] and Ruíz-Pérez et al. [6]. However, these arguments require further research, and this is the general purpose of our study in Ixtacxochitla, in central Mexico.

Given the harvesting and marketing strategies of the Ixtacxochitla community, we have asked how social, ecological, and economic factors interact with the traditional management of subsistence and commercially traded NTFPs, and how these factors drive the adoption of more or less intensive practices for commercially valuable species. We hypothesized that commercialized NTFPs will be managed more intensively than subsistence NTFPs. In addition, we expected that commercialized species that generate the highest net income and have the highest cultural value would be subjected to more intensive management practices, depending on other factors such as ecological and biological characteristics that may limit management to these species.

## Methods

### Study area

**Fig. 1:**
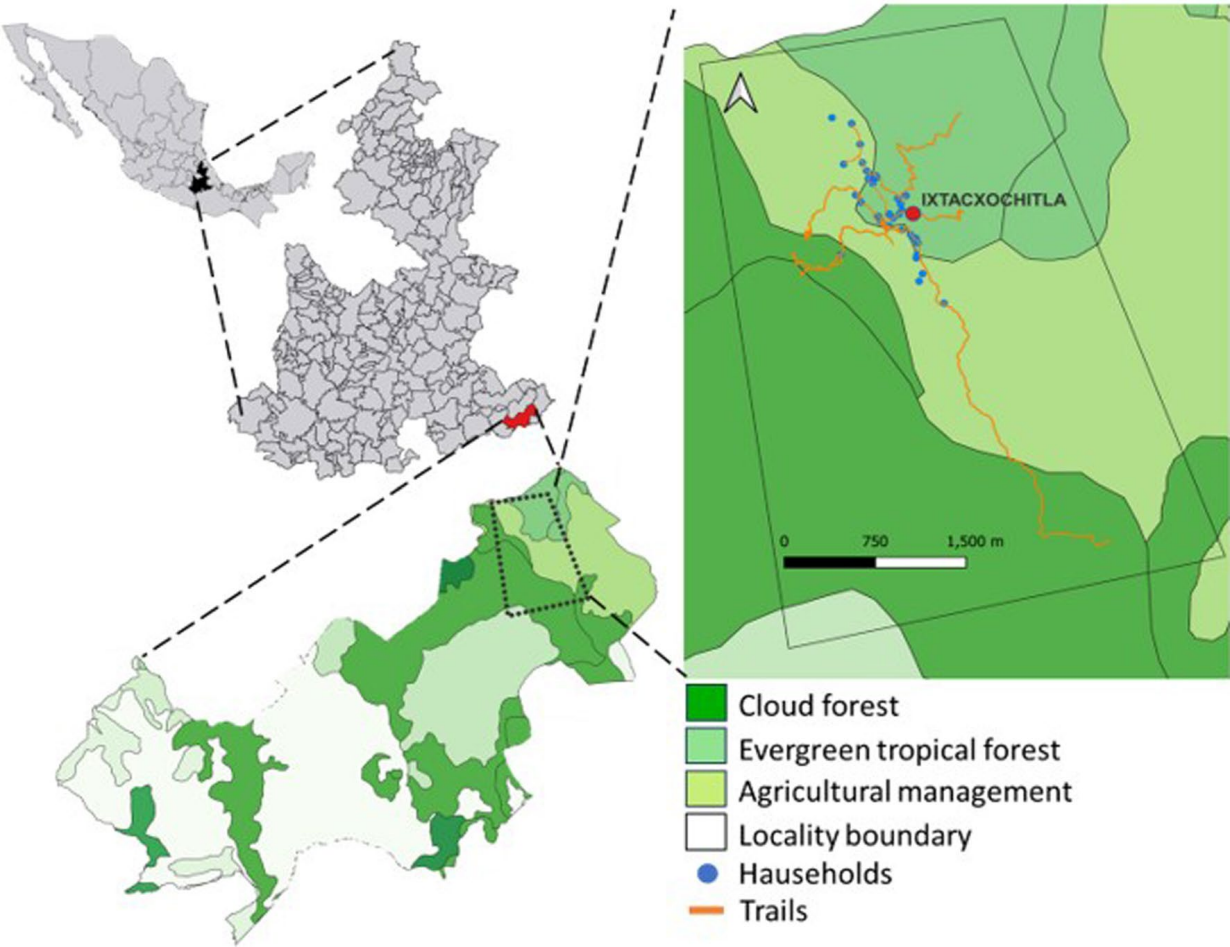
The community of Ixtacxochitla is located in the municipality of Coyomeapan in the state of Puebla, central Mexico

The study was conducted in the Sierra Negra, a mountainous region adjacent to the Tehuacán-Cuicatlán Valley, an area recognized for its significant ecological and cultural diversity [17, 35, 36]. Approximately 2700 plant species [37, 38] and eight cultural groups have interacted throughout the region for hundreds of years in the whole region [39]. Part of the mountainous region includes the Sierra Negra, where the locality of Ixtacxochitla is located, in the southwest of the municipality of Coyomeapan (Fig. 1) [38].

Ixtacxochitla is located at altitudes between 1100 and 2000 m, in a humid climate with summer rains, with annual precipitation ranging from 1500 to 2500 mm, and temperatures ranging from 14 to 20 °C. The vegetation in the area represents a transitional zone between tropical evergreen forests and mountain cloud forests [40–42]. Despite recent changes in land use for agricultural purposes, a significant portion (80%) of the forest cover has been preserved [41]. The community has a high level of marginalization and poverty, with a lack of basic services and access roads, which limits the socio-economic development of the 88 households and 402 inhabitants living in the community [43]. All residents are Nahua people and communicate among themselves primarily in their native language, although some are also fluent in Spanish.

The community is under ejidal ownership, with communal forest lands available to residents to extract natural resources for both commercial and subsistence purposes. These activities are regulated by community agreements that establish extraction rules [8, 42, 43]. The use of communal forest lands includes a variety of activities. The extraction of NTFPs is widespread among all families and serves as an important source of livelihood. A small proportion of the population participates in timber harvesting for construction purposes, meeting local infrastructure needs. In addition, the lower parts of the forest are used for slash-and-burn agriculture, resulting in a diverse landscape comprising areas with milpas (traditional practice of polyculture of maize *Zea mays*, and other crops), acahuales (secondary vegetation growing in fallow agricultural fields), and forest vegetation [42]. Subsistence agriculture is the main occupation of all families, while a small sector consisting of 9 households is engaged in the commercialization of various NTFPs to contribute to the household economy [31].

### Interviews and free listing

In 2019, we visited Ixtacxochitla to introduce the project and obtain the consent of the participants and local authorities to implement it. In 2022, we made visits to collect data on commercialization, plant management, and ethnobotanical collections. Before data collection, participants were selected using the non-probability snowball sampling method, in which interviewed persons recruit additional individuals among their acquaintances [44]. We included most people involved in the trade of NTFPs (9 participants from 7 houses), as well as people who used the products but did not sell them (21 participants from 21 houses). The 32% (28 houses) of all households in the community of Ixtacxochitla participated in the study, with an average household size of six members. The 73% of the interviewers were women (22 participants) and 27% (8 participants) were men, with an average age of 41 ± 14 years old. Although there are only 9 traders in the community, each one participates in a diverse trade encompassing up to 36 wild and managed species (see “Appendix 1”). This is a suitable sample for exploring variations in the management of NTFPs.

Initially, free listing interviews (referring to a list of items related to a specific topic) [44] were conducted to ask people about plants and mushrooms collected in the forest or nearby areas, that have some utility and emphasizing those with commercial value. Species names were recorded in both Nahuatl and Spanish, and any additional names were also documented. In addition, based on a literature review of factors influencing management practices [8–10, 14, 21], a semi-structured interview approach [44] was used to assess socio-ecological variables related to the sale and subsistence of NTFPs. Some examples of the general questions formulated were: Name the different places where you usually collect this plant/mushroom, How do you use this plant/mushroom? How many times a year do you use this plant/mushroom? What methods do you use to increase the establishment of this plant/mushroom? How abundant do you think this plant/mushroom is? Do you believe this plant/mushroom can grow outside its natural habitat through propagation and protection practices?

The specific variables assessed during the interviews included the following (Table 1):

- Use (u) denotes how people use a species;
- Frequency of consumption (fc) measures how often a product is used within a year;
- Management intensity (mi) indicates the degree of manipulation of the species;
- Cultural importance (ci) reflects values derived from the Relative Cultural Importance Index (RI) [45] explained below;
- Harvest sites (hs) refers to the zones where a species is harvested;
- Management feasibility (mf) refers to the participants' opinions on the possibility of establishing a species in ex situ environments through propagating and/or protection practices;
- Abundance perception (ap) refers to the participants' perceptions regarding the abundance of species and is determined by comparing three figures that varied in the number of plant images.

**Table 1:** Social, ecological, and economic factors influencing the management of NTFPs for commercialization and subsistence in the community of Ixtacxochitla

Factors influencing the management of NTFPs	Variable names	Variable abbreviation	Categories			
Sociocultural	Use	u	Ornamental	Edible	Medicinal	Ceremonial
	Frequency of consumption	fc	Annual/twice per year	Monthly	Twice per week	Weekly
	Management intensity	mi	Gathered	Tolerated	Propagated	Protected
	Cultural importance	ci	The value ranges from 0.1 to 0.8. Higher values correspond to greater cultural significance			
	Management feasibility	mf	Feasible	People perceived that plants can be established through propagation and protective practices		
			Not feasible	People perceived that plants cannot be established through propagation and protective practices		
			Without management	Propagation and protective practices have not been implemented		
Ecological	Harvesting system	hs	Forest	Ruderal	Acahual	Milpa
			Coffee plantation		Homegarden	
	Perception of abundance	pa	Abundant	The interviewees selected the figure displaying 100% of the area covered by plant images		
			Regular	The interviewees selected the figure displaying 50% of the area covered by plant images		
			Scace	The interviewees selected the figure displaying 15% of the area covered by plant images		
Commercial	Annual net income	*u*_*i*_	Net income from commercialization of the NTFPs, estimated in Mexican pesos per year (MXN/year), with a mean exchange rate of 1 United States dollar (USD) ≈ 17.3 Mexican pesos (MXN) in January 2024			

Finally, semi-structured interviews were also conducted with traders at the Coyomeapan market to assess economic variables of the commercialized species; the species mentioned by each trader are observed in “Appendix 1.” Some of the general questions formulated to the traders include: What is the total cost of selling this species? How much does this species cost? How often do you come to sell at the market? In which months is this species available for sale? In addition, the commercialized mass in kilograms for each species was quantified. This information was useful for the development of a cost–benefit model to calculate the net income of each commercialized species. All the interviews and the free lists were conducted in Spanish, although in some cases an interpreter was needed to translate from Nahuatl to Spanish and vice versa.

### Ethnobotanical collections and taxonomic identification

Ethnobotanical studies were carried out to collect botanical specimens under the collection number SGPA/DGGFS/712/2918/18 authorized by the Secretaría del Medio Ambiente y Recursos Naturales. The collected specimens were identified using checklists previously developed for the study area [17, 31, 33, 34, 46] and supplemented by the use of botanical keys [47, 48]. The voucher specimens were deposited at the National Herbarium of Mexico (MEXU). Of the 57 plant species collected, 52 were determined at the species level, four were classified only at the genus level, and one at the family level. Of the seven fungal species collected, four were identified at the species level and three at the genus level with the help of a specialist. Unidentified plants with some usefulness were considered as “ethnospecies” for the analyses performed.

### Data analyses

#### Estimating the cultural importance index

The Relative Cultural Importance Index (RI) proposed by Pardo de Santayana [45] is based on the degree of agreement among informants regarding the utility of species in relation to the diversity of uses. This index is based on the premise that the more important a species is, the more likely it is to be mentioned and the greater the number of uses associated with it [49]. The RI was calculated as follows:

$$\normalsize {\text{RI}}_{{\text{s}}} = \frac{{{\text{RFC}}_{{{\text{s}}\left( {\max } \right)}} + RNU_{{{\text{s}}\left( {\max } \right)}} }}{2}$$

where RI_s_: Relative Cultural Importance Index for a species s; RFC_s(max)_: relative mention frequency of a species compared to the maximum number of informants mentioning that species; RNU_s(max)_: relative number of use categories compared to the maximum number of use categories for all species.

Higher values obtained from the RI indicate the species that are more frequently mentioned and have greater use categories, while lower values represent species that are less frequently mentioned and have specific uses according to respondents [45].

#### Socio-ecological factors in NTFPs management for subsistence and commercialization

To understand the relationships between socio-ecological variables and the management practices of NTFPs used for commercialization and/or subsistence, a non-metric multidimensional scaling multivariate analysis (NMDS) was used. The input matrix for this analysis included all variables except annual net income from Table 1. To determine the fit of the data, the stress value generated by the model was used, with values below 0.25 indicating a higher explained variance of the data in the space [50].

Nonparametric tests were used to determine the statistical difference between the socio-ecological variables analyzed for subsistence and commercialization. For six categorical variables (u, hs, pa, mi, mf, and fc), a Chi-squared test followed by Haberman's corrected residuals test was used. The aim was to identify differences between the categories within each variable for NTFPs for subsistence and commercialization. For the numerical variable “cultural importance” (ci), normality and homogeneity of variance tests were performed, but since they did not meet the assumptions, the nonparametric U-Mann–Whitney test was used [51, 52].

#### Cost–benefit analysis for assessing the commercial net income from NTFPs commercialized

A cost–benefit model was developed to estimate the net income from the commercialization of each species traded in the regional market of Coyomeapan [53]. The purpose was to analyze the potential impact of income on management intensification. The development of the cost–benefit model required an examination of the activities of the traders throughout the commercialization process. The collection of the NTFPs usually takes place the day before the Sunday market. The traders have to collect the species in the nearby forest, which is usually more than 5 km away from Ixtacxochitla, and also from the agroforestry systems, which are located between 0 and 2 km, depending on the species. The harvesting time needed varies depending of the species, but it can take all day long. For longer distances, the traders take the products back on their own mules, usually in raffia sacks. Back in the village, the products are packed for further transport in wooden crates or in bast sacks, which are reused for each market day. On the market day (Sunday), the products have to be transported in the morning first on a dirt road for about 3 km. The trader is accompanied by another family member and their mule. Afterward, the trader takes the products on a van that is rented among several traders, on the 32 km long road to the market in Coyomeapan. At the market, the trader has to pay a fee to occupy a space and then stays all day (about 8 h). At the end of the day, the trader takes the same van back and then walks the 3 km back to the village. Unsold NTFPs are left with acquaintances near the market at no extra cost until the next market day. Each trader commercializes a variety of species, ranging from four to 32, depending on their seasonal availability (“Appendix 1” lists the species mentioned by each trader and Table 4 shows the seasonal availability by species).

To obtain the value of the net income per kilogram of a given species, we took into account the monetary costs of packaging materials and transportation, but not the time invested. The costs did also not include the mules used to transport the goods, since these animals live on the family’s property and eat natural vegetation.

The annual net income of each species was calculated with the following formula:

1 $$u_{i} = \left( {p_{i} - \frac{{\left( {c_{{\text{t}}} + c_{v} } \right) \cdot n}}{{\mathop \sum \nolimits_{i = 1}^{k} [m_{i} \cdot d_{i} ]}}} \right) \cdot m_{i} \cdot d_{i} ,$$

where *c*_t_: cost of transportation in a van (round trip between Ixtacxochitla and the market in Coyomeapan) and wooden crates per day of sale, *c*_*v*_: daily charge for renting a space in the market for the sale of the product, *d*_*i*_: number of days during in a year when the product of species *i* is available for sale, *k*: number of species of NTFP thar are commercialized in 1 year, *m*_*i*_: mass in kilograms of species* i* harvested for 1 day on the market, *n*: number of days a trader is at the market in a year, *p*_*i*_: selling price per kilogram for species *i*, and *u*_*i*_: annual net income per species.

Formula (1) calculates in brackets the price (*p*_*i*_) minus the cost per kilogram for a given species and then multiplies the net income per kilogram by the mass sold per day (*m*_*i*_) and by the number of days that the product is sold on the market per year (*d*_*i*_). The only part that requires explanation is the cost per kilogram. This unit cost represents an average for all NTFPs, since all expenses related to transportation, packaging, and market fees, which remained constant throughout the year, were shared among the different species mixes. Consequently, the ratio represents the cost for the trader to attend the market during a year, divided by the total quantity (mass) of the NTFPs for all species brought to the market during that year. The costs *c*_*t*_ and *c*_*v*_ were 120 MXN (6.94 USD) and 3 MXN (0.17 USD), respectively. Data for the remaining variables for each species are presented in Table 4. For example, the annual net income from edible inflorescences of the *Chamaedorea tepejilote*, was calculated as follows:

$$\begin{aligned} u_{i} & = \left( {15\;{\text{MXN}}/{\text{kg}} - \frac{{\left( {120\;{\text{MXN}}/{\text{d}} + 3\;{\text{MXN}}/{\text{d}}} \right) \cdot 48\;{\text{d}}/{\text{a}}}}{{3493\;{\text{kg}}/{\text{d}}}}} \right) \cdot 12\;{\text{kg}}/{\text{d}} \cdot 48\;{\text{d}}/{\text{a}} \\ & = 7666\;{\text{MXN}} \\ \end{aligned}$$

#### Economic and socio-ecological factors in management of commercialized NTFPs

Principal component analysis (PCA) [50] was used to examine the relationship between NTFPs management intensity and economic and socio-ecological factors. Only commercially traded products were included in this analysis, as these are the products that generate quantifiable economic incomes. The input matrix for the PCA was constructed using five variables (mi, ci, pa, *u*_*i*_, and *m*_*i*_) from Table 4, as they provided the most accurate explanation for the ordination of the data. All multivariate analyses and significance tests were performed using the R software version 4, using the “FactoMineR” package for multivariate analyses.

## Results

In Ixtacxochitla, 64 species of NTFPs are harvested, of which 44% (28 species) are used for subsistence and 56% (36 species) are both subsistence and commercialized in the municipal market of Coyomeapan.

The species used for subsistence (*S*) and commercialization (*C*) have different socio-cultural characteristics in terms of use, frequency of consumption, intensity of management, cultural importance, and feasibility of management. They also differ in terms of the systems in which they are harvested and the perceived abundance of the resource according to the respondents (Table 4 describes the socio-ecological variables of commercialized species, while “Appendix 2” details those of subsistence species). Both types of species are managed at different intensities, through (1) incipient in situ gathering practices within forests. (2) Intermediate practices include the tolerance of species in anthropogenic areas such as roadsides, acahuales (fallow fields), and milpas (cornfields). (3) Intensive protection practices include weeding, pruning, and insect pest control or elimination; and (4) actions that promote the presence of species in ex situ environments, such as seedlings/young plants transplantation or seed dispersal in homegardens, coffee plantations, and milpas. Certain species are managed using a single practice, while others are managed using a combination of practices. Finally, a greater proportion of subsistence species are perceived as abundant, as opposed to commercialized species, which are perceived as scarce (see Table 2). The management and trade of NTFPs are primarily linked with women, while collection is associated with men, especially when carried out in remote forest areas.

**Table 2:** Proportion of subsistence (*S*) and commercialized (*C*) species comprising socio-ecological variables evaluated in the community of Ixtacxochitla

Factors influencing the management of NTFPs	Variable names	Categories	*S* (%)	*C* (%)
Sociocultural	Use	Ornamental	22	5
		Edible	25	71
		Medicinal	34	14
		Ceremonial	19	10
	Frequency of consumption	Annual/twice per year	64	30
		Monthly	14	10
		Twice per week	7	23
		Weekly	14	38
	Management intensity	Gathered	20	24
		Tolerated	48	28
		Protected	11	18
		Propagated	24	30
	Feasibility of management	Feasible	79	75
		Not feasible	3	6
		Without management	18	19
	Cultural importance		0.21	0.34
Ecological	Harvesting system	Forest	22	24
		Ruderal	37	26
		Coffee plantation	6	14
		Homegarden	18	19
		Acahual	13	6
		Milpa	3	11
	Perception of abundance	Abundant	44	26
		Regular	28	26
		Scace	28	47

### Socio-ecological factors in NTFPs management for subsistence and commercialization

**Fig. 2:**
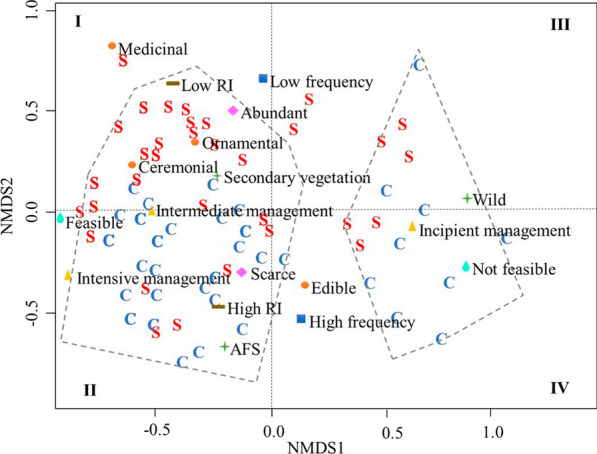
Non-metric multidimensional scaling to examine the arrangement of subsistence (*S*) and commercialized (*C*) species in terms of the management intensity, uses, perceived abundance, frequency of consumption, cultural importance, and management feasibility. The categories for each variable are: low frequency (annual/twice per year consumption), high frequency (weekly/twice per week consumption), intensive management (protected and propagated), incipient management (gathered), intermediate management (tolerated), AFS: agroforestry systems (coffee plantation, milpa, home garden), secondary vegetation (acahual, ruderal), wild (cloud forest and tropical forest), low RI (*x*^2^ = 0.21 ± 0.13), high RI (*x*^2^ = 0.34 ± 0.16). The gray-colored polygons show how the management feasibility variable affects species composition; on the left are species that are considered manageable, while on the right are those that are not. The model produced a “stress value” of 0.21, suggesting a relatively satisfactory dispersion of the data in space

The relationship between socio-ecological variables and management practices for subsistence (*S*) and commercialized (*C*) species is examined using non-metric multidimensional scaling (NMDS) (Fig. 2). The results show a segregation of subsistence and commercialized species clustering of subsistence species for at the top of the plot, while commercialized species are at the bottom. In addition, the plot shows a segregation of species from right to left based on the variable feasibility of management. Species that people perceive to be unmanageable outside their natural habitat are placed on the right side, while those manageable in anthropogenic environments are placed on the left. The variables influencing the distinction between subsistence and commercialized species included a significant increase in subsistence species in the case of medicinal use, while edible species are predominantly traded. Additionally, commercialized species show significantly higher values in both frequency of consumption and cultural importance index compared to subsistence species (Table 3 shows the significance values of these variables).

**Table 3:** Socio-ecological variables associated with the separation of subsistence and commercialized species

Variable	Statistic test	Statistic value	*P* value
Use	*X*^*2*^	16.32	0.0009
Frequency of consumption	*X*^*2*^	12.25	0.01
Cultural importance	Mann–Whitney U	281	0.000

The species in quadrant I of the plot are mainly NTFPs used for subsistence, characterized by receiving practices of intermediate management intensity, such as tolerance in secondary vegetation zones. They are mainly used for medicinal purposes, such as *Ocimum micranthum* (Clavoxivitl) and *Sambucus nigra* (Xómet), while other species have ornamental functions such as *Verbesina turbacensis* (Zazastli) and ceremonial uses such as *Begonia heracleifolia* (Cecigxochitl). These plants are perceived as abundant, are used infrequently (twice a year), have low cultural importance value, according to the CI index, and are accessible resources that are collected when needed.

The species in quadrant II are primarily those that are commercialized, although some species are for subsistence. In both cases these species are subject to more intensive management practices. For example, the inflorescences of *Chamaedorea tepejilote* (Tepejilote) are collected from forest populations, although seed dispersal and protection are also observed in agroforestry systems such as coffee plantations and homegardens. Similarly, species such as *Cestrum nocturnum* (Zopequilitl), *Solanum americanum* (Tomaquilitl), and *Porophyllum ruderale* (Pápalo) are cultivated and protected in homegardens, although they are also tolerated in milpas, acahuales, and other agroforestry systems. The species *Talauma mexicana* (Yoloxochitl) is managed through gathering and propagation, while *Litsea glaucescens* (Laurel) is managed through gathering and tolerance.

In quadrant II, commercialized species are mainly used for food purposes, although some are also used for medicinal and ornamental purposes. These species are perceived as less abundant (low or regular), have a higher frequency of extraction (twice per week or weekly), and have higher cultural importance values. For subsistence species positioned in this quadrant, the practices were more intensive compared to those in quadrant I, as was the case of *Witheringia solanacea* (Xaltojto) and *Spathiphyllum wallisii* (Ixtacxochitl), for which propagation and protection actions were indicated to be carried out in homegardens.

The species not considered feasible for management (quadrants III and IV) are different from those in quadrants I and II, because they are wild species collected from cloud forests and tropical forests that are more distant and require more time to harvest.

Quadrant III includes products of wild species for subsistence (*S*), which are used for ornamental and edible purposes, collected sporadically (twice a year) and of low cultural importance, such as *Epiphyllum ackermannii* (Papaloxochitl) and *Amanita rubescens s.l*. (Xochitegonsi). Quadrant IV includes wild species of commercial value, such as *Peperomia peltilimba* (Tequelite), *Arisaema macrospathum* (Nechigolispactli) and mushrooms, such as *Laetiporus gilbertsonii* (Chilanancatl) and *Auricularia auricula-judae* (Tonagaz). These species are perceived as more abundant among the commercialized species and, similar to the species in quadrant II, they are used as food, have a high frequency of extraction (twice a week or weekly) and high cultural importance values.

### Net income from the commercialization of NTFPs

**Fig. 3:**
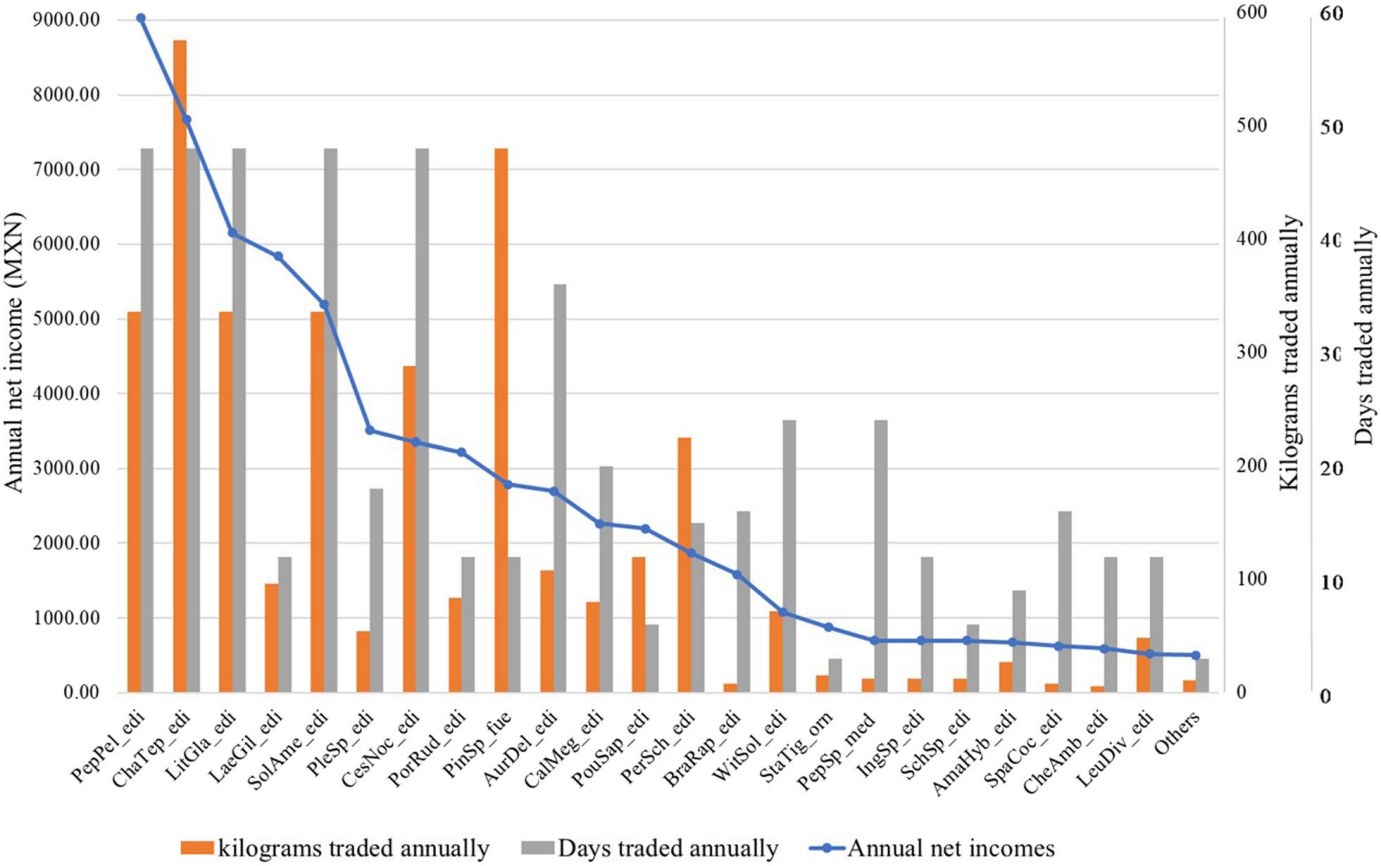
Variations in annual net income, kilograms traded, and number of days traded annually among the 36 species commercialized in the regional market of Coyomeapan. The species abbreviations consist of the first three letters of the genus and species name, followed by the three letters of the use category. The species are: PepPel: *Peperomia peltilimba*, ChaTep: *Chamaedorea tepejilote*, LitGla: *Litsea glaucescens*, LaeGil: *Laetiporus gilbertsonii*, SolAme: *Solanum americanum*, PleSp: *Pleurotus* sp., CesNoc: *Cestrum nocturnum*, PorRud: *Porophyllum ruderale*, PinSp: *Pinus* sp., AurDel: *Auricularia delicata*, CalMeg: *Calyptranthes megistophylla*, PouSap: *Pouteria sapota*, PerSch: *Persea schiedeana*, BraRap: *Brassica rapa*, WitSol: *Witheringia solanácea*, StaTig: *Stanophea tigrina*, PepSp: *Peperomia* sp., IngSp: *Inga* sp., SchSp: *Schizophyllum* sp., AmaHyb: *Amaranthus hybridus*, SpaCoc: *Spathiphyllum cochlearispathum*, CheAmb: *Chenopodium ambrosioides*, LeuDiv: *Leucaena diversifolia*. The uses categories are: edi: edible, orn: ornamental, med: medicinal, fue: fuel

The cost–benefit analysis indicated that the sum of the annual income generated by each of the 36 commercialized NTFPs resulted in a total annual net income of 67,526 MXN (about 3924 USD) per household involved in commercialization in Ixtacxochitla. Five species generated higher net incomes, ranging from 5000 MXN to 9000 MXN (293 USD to 529 USD) per species per year: *Peperomia peltilimba* contributes 13.4% of the total annual income, followed by *Chamaedorea tepejilote* with 11.4%, *Litsea glaucescens* with 9.1%, *Laetiporus giltbersonii* with 8.6%, and *Solanum americanum* with 7.7%. These five species are also characterized by the considerable quantity commercialized annually (more than 100 kg/year), the extensive periods available for marketing (more than 48 weekends per year) (Fig. 3, Table 4), and being the most frequently chosen by the traders.

**Table 4:** Socio-ecological and economic variables examined for the 36 NTFPs commercialized in the Coyomeapan market

Specie	Use	frec. con	man. int	man. fea	cul. imp	harv. sys	per. abu	net uti		pri. kg	mass kg	days ava	Months
								MXN	%				
*Peperomia peltilimba*	E	W	G	NF	0.5	F	R	9032	13	29	336	48	AMY
*Chamaedorea tepejilote*	E,C	W	G,P,PG	F	0.8	F,R,H,F,C	A	7666	11	15	576	48	AMY
*Litsea glaucescens*	E,M,C	W	G,T	F	0.5	F,R	S	6152	9	20	336	48	AMY
*Laetiporus gilbertsonii*	E	W	G	WM	0.5	F	R	5838	9	63	96	12	A,Ma,A,S
*Solanum americanum*	E	W	T,P,PG	F	0.6	H,M,A	R	5192	8	17	336	48	AMY
*Pleurotus sp.*	E	W	G	WM	0.5	M,C	S	3509	5	67	54	18	A,Ma,Jn,Jl,A,S
*Cestrum nocturnum*	E	W	T,P,PG	F	0.6	H,M,C	S	3353	5	13	288	48	AMY
*Porophyllum ruderale*	E	W	P,PG	F	0.2	M,C,A	A	3218	5	40	84	12	Jn,Jl,A
*Pinus sp.*	F	M	G	WM	0.2	F	R	2789	4	8	480	12	AMY
*Auricularia delicata*	E	W	G	WM	0.4	F,R,M,C	R	2697	4	27	108	36	AMY
*Calyptranthes megistophylla*	E	W	G,T,PG	F	0.3	F,R,H,F	R	2265	3	30	80	20	Ma,Jn,Jl,A,S
*Pouteria sapota*	E	B	G,T	F	0.2	R	S	2197	3	20	120	6	A,Ma,Jn
*Persea schiedeana*	E	W	T,P,PG	F	0.2	R,H	S	1870	3	10	225	15	A,Ma,Jn,Jl, A
*Brassica rapa*	E	B	P,PG	F	0.3	M	S	1586	2	20	8	16	Ma, Jn, Jl, A,S,O,N,D
*Witheringia solanacea*	E	B	T,P,PG	F	0.5	R,H,C,A	S	1078	2	17	72	24	AMY
*Stanophea tigrina*	O	A-S	G,P,PG	F	0.5	H,F	S	875	1	60	15	3	A,Ma,Jn
*Inga sp.*	E	M	T,PG	F	0.2	R,H	A	700	1	60	12	12	Jn,Jl,A,S
*Peperomia sp.*	M	B	PG	F	0.2	F,R,H	A	700	1	60	12	24	AMY
*Schizophyllum comune*	E	B	G	WM	0.2	R,A	A	700	1	60	12	6	Ma,Jn,Jl
*Amaranthus hybridus*	E	W	T,P,PG	F	0.3	H,M	A	674	1	27	27	9	Ma,Jn,Jl
*Spathiphyllum cochlearispathum*	E	B	G,T,PG	F	0.4	F,R,H,C	S	626	1	80	8	16	Ma, Jn, Jl, A,S,O,N,D
*Chenopodium ambrosioides*	E	B	T,P,PG	F	0.3	R,H	R	590	1	100	6	12	Jn,Jl,A,S,O
*Leucaena diversifolia*	E	W	T	F	0.4	F,R,C	R	519	1	13	48	12	Jn,Jl,D,Ja
*Taluma mexicana*	E,M,C	B	T	F	0.7	F,R,C	R	515	1	12	50	2	Ma,Jn
*Diospyros nigra*	E	A-S	T	F	0.3	R	S	483	1	50	10	1	Ma
*Peperomia maculosa*	E	M	G,PG	F	0.2	F,R	S	366	1	20	20	2	Ja,D
*Renealmia alpinia*	E	A-S	P,PG	F	0.2	R,H	S	350	1	60	6	6	Ja, M,Ma,Jl,S,N
*Tagetes erecta*	C	A-S	PG,PG	F	0.2	R,H,A	S	303	0	32	10	2	O
*Agave obscura*	E	A-S	G	WM	0.3	F	A	297	0	200	1.5	3	A,Ma,Jn
*Chamaedorea tepejilote*	E,C	W	G,P,PG	F	0.8	F,R,H,F,C	A	266	0	15	20	1	A
*Arisaema macrospathum*	M	M	G	NF	0.2	F	A	246	0	100	2.5	5	A,Ma,Jn,O,N
*Cedrela oaxacensis*	M	A-S	T	F	0.2	F,R,C	S	233	0	60	4	4	AMY
*Etno b*	E	A-S	G	WM	0.3	F	A	197	0	100	2	2	Ja,F,M
*Begonia caroliniifolia*	E	A-S	T,PG	F	0.2	F,M	S	140	0	13	12	4	F,A,Jn,A
*Canna indica*	O	A-S	T,P,PG	F	0.3	R,H	R	135	0	16	9	3	Jn,Jl,A
*Pouteria campechiana*	E	A-S	T	F	0.2	R	S	110	0	20	6	1	Ma
*Etno a*	M	A-S	T,PG	F	0.2	R,H	S	58	0	60	1	1	AMY

Use: O, Ornamental; E, Edible; M, Medicinal; C, Ceremonial; frec. con. (consumption frequency): A, Annual/Twice per year; M, Monthly; B, Twice per week; W, Weekly; man. int. (management intensity): G, Gathered; T, Tolerated; P, Protected; Pr, Propagated; man. fea. (management feasibility): F, Feasible; NF, Not feasible; WM, Without management; cul. imp. (cultural importance); har. sys. (harvesting system): F, Forest; R, Ruderal; A, Acahual; M, Milpa; C, Coffee plantation; H, Homegarden; per. abu. (abundance perception): A, Abundant; R, Regular; S, Scace; net uti. (annual net incomes); pri. kg (selling price per kilogram); mass kg (mass in kilograms harvested); days ava. (number of days the product is sold annually). Months:AMY, All months of the year; Ja, January; F, February; M, March; A, April; Ma, May; Jn, June; Jl, July; A, August; S, September; O, October; N, November; D, December

Intermediate contributions between 1000 MXN and 3500 USD (58 USD to 203 USD) per species and year were observed for ten other species, while the remaining 22 species generated annual net incomes below 1000 MXN (58 USD) each one. Net incomes and commercialized quantities per species decreased with naturally shorter periods of availability of NTFP during the year (Fig. 3, Table 4).

### Economic and socio-ecological factors in the management of commercialized NTFPs

**Fig. 4:**
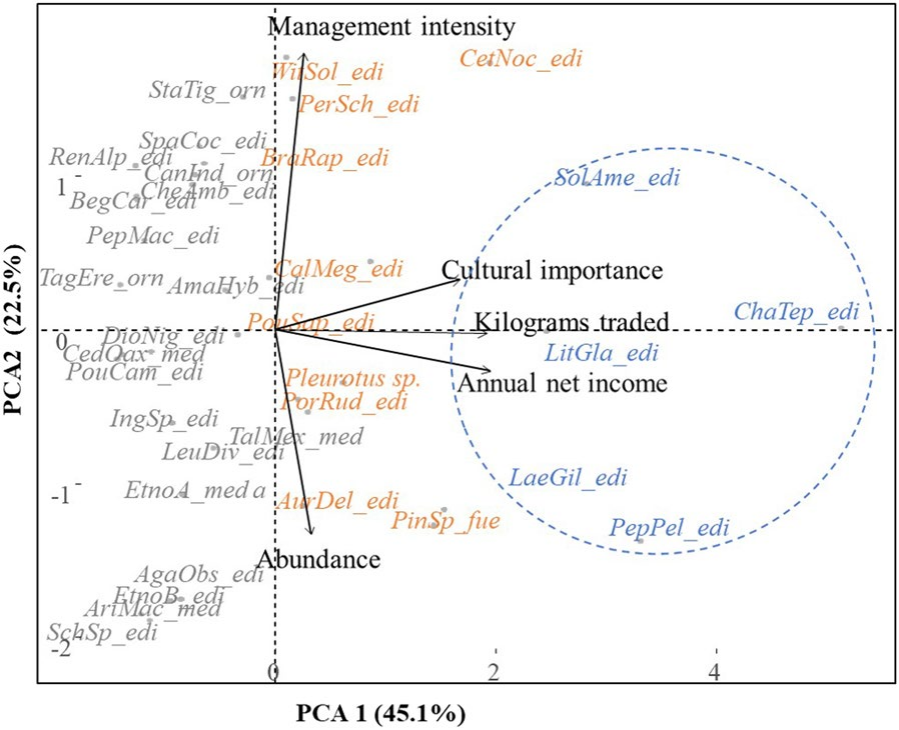
Principal component analysis conducted for the socioecological and economic variables associated with the management of commercialized NTFPs. The five species within the blue circle are the ones with the highest cultural value, the highest number of kilograms traded annually, and the highest annual net income. The species at the top of the graph are more intensively managed, while the species with the highest abundance are at the bottom*. PepPel*: *Peperomia peltilimba, ChaTep*: *Chamaedorea tepejilote, LitGla: Litsea glaucescens, LaeGil: Laetiporus gilbertsonii, SolAme: Solanum Americanum, PleSp: Pleurotus sp., CesNoc: Cestrum nocturnum, PorRud: Porophyllum ruderale, PinSp: Pinus sp., AurDel: Auricularia delicata, CalMeg: Calyptranthes megistophylla, PouSap: Pouteria sapota, PerSch: Persea schiedeana, BraRap: Brassica rapa, WitSol: Witheringia solanácea, StaTig: Stanophea tigrina, PepSp: Peperomia sp., IngSp: Inga sp., SchSp: Schizophyllum sp., AmaHyb: Amaranthus hybridus, SpaCoc: Spathiphyllum cochlearispathum, CheAmb: Chenopodium ambrosioides, LeuDiv: Leucaena diversifolia, TalMex: Taluma mexicana, DioNig*: *Diospyros nigra, PepMac: Peperomia maculosa, RenAlp: Renealmia alpinia, TagErc: Tagetes erecta, AgaObs: Agave obscura, AriMac: Arisaema macrospathum, CedOax: Cedrela oaxacensis, BegCar: Begonia caroliniifolia, CanInd: Canna indica, PouCam: Pouteria campechiana, EtnoA: Tonalxivitl, Etno b: Alactzin.* The uses categories are: edi: edible, orn: ornamental, med: medicinal, fue: fuel

The results of the PCA in Fig. 4 show the relationship between the management intensity of the commercialized species and the economic and socio-ecological factors. Based on the analysis, the first two principal components explain 67% of the variance of the data, with the first component (PC1) explaining 45%, and the second component (PC2) 22%, together representing 77% of the explained variance. The eigenvalues indicated that the variables with the greatest influence on the ordination of the commercialized NTFPs were the annual net income for PC1 and the management intensity for PC2 (Fig. 4).

According to PC1, species with the highest annual economic income are clustered on the right side of the graph, highlighted in blue in Fig. 4. These species also have the highest extraction rates in terms of kilograms, are the most frequently sold throughout the year, have the highest cultural importance values, and are perceived to have regular to rare in abundance. Toward the left side of the graph, there is an orange cluster of species, characterized by intermediate values of net income, extraction rates, cultural importance, sales frequency, and perceived abundance. The species in gray represent those with the lowest values in these variables, except for abundance.

The second principal component (PC2) illustrates an arrangement of species with more intensive management practices positioned at the top of the graph, while those with lower levels of management are at the bottom (Fig. 4). Species with more intensive management are those to which people apply multiple practices to tolerate, propagate, and protect in agroforestry systems such as *Solanum americanum*, *Cestrum nocturnum*, *Witheringia solanacea*, *Amaranthus hybridus* (Bahaquilitl), *Porofilum ruderale*, *Brassica rapa* (Colesh), *Chamaedorea tepejilote*, among others. These species are followed by others that receive intermediate practices of tolerance and gathering, represented by *Litsea glaucescens*, *Leucaena diversifolia* (Guaje), *Taluma mexicana*, and *Calyptranthes megistophylla* (Mototl). Finally, we identify species whose products are obtained through simple gathering practices within the forest, such as *Peperomia peltilimba*, *Laetiporus giltbersonii*, *Pleurotus* sp. (Iztanancatl), *Pinus* sp. (Ocote), *Arisaema macrospathum*, among others.

In Fig. 4, the five species with the highest net incomes are grouped in an oval, to highlight that these species are subject to gathering practices, as observed in *Peperomia peltilimba* and *Laetiporus giltbersonii*, intermediate practices, as in *Litsea glaucensens*, and more intensive practices as in *Chamaedorea tepejilote* and *Solanum americanum*.

## Discussion

### Diversification of management practices of NTFPs for subsistence and commercialization

According to our initial hypothesis, it was expected that commercialized NTFPs would exhibit more intensive management practices than those intended for subsistence. However, the management strategies for species in both groups turned out to be more complex than expected. The commercialized products, consisting mainly of edible species and some ceremonial species, show a gradient of practices ranging from incipient to intensive. Edible and ornamental subsistence species are managed through intensive practices, while medicinal subsistence species tend to intermediate tolerance practices, and some ornamental species tend to incipient gathering practices. In this context, it can be argued that the commercial importance of a resource per se does not necessarily imply the intensification of management practices.

Species management studies have recognized that a critical factor influencing the management intensification is the biological feasibility of species to be established in anthropogenic environments [13, 21, 26]. Species with physiological traits that allow them to maintain their life cycle and reproductive system in environments different from their natural habitat, are more likely to undergo intensive practices to enhance resource availability [8, 21, 25, 26]. Similarly, the biological feasibility of manipulating species was a key factor in the management decisions articulated by the people. Species for both subsistence and commercial use were managed through intensive practices when people perceived biological feasibility for their management.

Other factors that synergized with management feasibility included the ecological abundance of the species, the frequency of consumption, and the cultural importance of each species. Together, these aspects played a role in determining the people’s adoption of more or less intensive management practices. For example, with respect to medicinal plants for subsistence, factors that may have influenced the adoption of practices characterized by tolerance in ruderal zones are sporadic consumption in the locality, the perception of abundance in their environment, and the low cultural value attributed to them. Similar management approaches for subsistence medicinal species have been observed in other regions, where occasional and limited use leads to the adoption of intermediate management practices [14]. One reason for this is that in situations where species are highly available and extraction rates are low, the risk of resource depletion decreases, thus reducing the need for employ-intensive strategies aimed at increasing their availability [17, 21, 26].

The most intensive management strategies were observed for edible and ornamental NTFPs intended for both subsistence and commercialization. In the case of ornamental plants, the sense of well-being that they provide to people by embellishing homes, communal spaces, and places of worship encourages the adoption of intensive practices to ensure their acquisition, especially if they have cultural value [14, 18]. As suggested by Blancas et al. [8], Rangel-Landa et al. [14], and Larios et al. [34]*,* the management of ornamental species is primarily influenced by their aesthetic value and cultural significance, rather than uncertainties about their availability as observed for edible resources. In this context, it is understandable that in Ixtacxochitla, the ornamental species *Spatthiphullum wallisii*, *Stanhopea tigrina* (Tehuxochitl), and *Sobralia macrantha* (Tzinagaxochitl), which are widely used and culturally valued for garden and interior decoration, were managed through propagation in homegardens.

For edible species, the concern to ensure the availability of resources to meet basic food needs drives the intensification of management efforts and the increase in their production [13, 17, 24, 26], especially when they are culturally important species that tend to be more consumed and therefore less available [14, 54]. In our research we found similarities in these aspects: edible plants for commercialization and subsistence that received intensive protection and propagation practices, were those with high cultural value, were frequently consumed, were perceived as scarce in their natural environment, and feasible for management in ex situ environments. Outstanding products are some “quelites” (tender edible vegetables of several species) and species with edible fruits or flowers, such as *Calyptranthes megistophylla*, *Persea schiedeana* (Chinene), *Pouteria sapota* (Mamey), *Spathiphyllum cochlearispathum* (Ixtacxochitl), and *Chamaedorea tepejilote*. In other rural regions, these species are also managed to increase their availability and consumption due to their nutritional importance [2, 31, 55].

Edible NTFPs used for subsistence versus commercialization showed some differences in their management strategies. For subsistence, management focused on propagation of species exclusively in homegardens, while species with commercial value were managed across different types of agroforestry systems. Species such as *Cestrum nocturnum*, *Witheringa solanacea*, and *Chamaedorea tepejilote*, are managed in homegardens to ensure their availability for subsistence. However, when they are commercialized, their growth is propagated in coffee plantations, milpas, acahuales, as well as ruderal vegetation sites. It has been proposed that the increase in demand for a resource due to its commercial value may lead to adjustments in management strategies to improve production [16]. The diversification of harvesting sites for some commercialized NTFPs in Ixtacxochitla is likely a strategy implemented in response to market demand to increase the availability of commercially valuable species.

Other edible NTFPs feasible for management under less intensive practices include *Litsea glaucescens*, *Taluma mexicana*, and *Litsea diversifolia*. Despite their regular trade and cultural value, the management of these species relies on the tolerance of individuals in *ex situ* environments. Casas et al. [12, 25] suggest that slow-growing species, such as those mentioned above, are managed through tolerance in agroforestry systems or ruderal vegetation areas, since the benefits of intensified management are not available in the near future.

The NTFPs with the most incipient management practices were those directly gathered from their wild habitats, including both subsistence and commercial species. Subsistence products include edible fungi that meet nutritional needs and ornamental plants that improve the quality of life. They share characteristics such as low cultural value, low consumption (once a year), and high abundance in their natural environment. These species have received incipient management due to the certainty of their availability [8, 12] and their limited consumption [54].

NTFPs destined for commercialization and management through simple gathering include edible plants and mushrooms with high cultural value, that are widely consumed, but lack the feasibility of management. This phenomenon is evident in the mushrooms *Laetiporus giltbersonii, Pleurotus* sp., and *Auricularia* sp. (Tonagaz), which require specific substrates for growth in forest environments, making them difficult to propagate [56]. The same challenge applies to *Peperomia peltilimba*, a “quelite” that has been attempted to propagate in Ixtacxochitla and other localities by transplanting from its natural habitat to family homegardens, but without success [31, 57, 58]. These results support the idea proposed by Blancas et al. [8], Casas et al. [25], and Delgado-Lemus et al. [26], suggesting that regardless of the cultural value and level of consumption of a species, the possibility of intensifying its management will depend on the feasibility for management and adaptation to human-made environments. Therefore, species with limited adaptability to anthropogenic environments will be subject to incipient management practices such as gathering [54].

Other factors influencing the adoption of more intensive management practices in rural areas include the secure land tenure that allows communities to make long-term decisions [5, 9, 16]. In addition, technological support, access to agricultural services, and the presence of government programs that promote the development of appropriate infrastructure to enhance resource production may be important [2, 8, 59]. In Ixctacxochitla, the lack of such external support to create the conditions for intensive management is a constraint to intensified production.

### How commercial value influences NTFPs management decisions

The literature suggests that when an NTFP generates substantial economic benefits, management efforts are generally intensified, to improve production and increase sales [5, 7]. In this context, our initial hypothesis was that NTFPs with the highest economic returns would be the most intensively managed. However, our results from Ixtacxochitla showed no clear pattern of increased management effort for NTFPs with high net income. Species with high and low commercial returns were managed with a wide range of practices, from incipient to intensive. Even the five species with the highest commercial returns were managed across this gradient of practices including simple gathering (*Peperomia peltilimba* and *Laetiporus giltbersonii*), tolerance (*Litsea glaucescens*), and intensive protection and propagation practices (*Chamaedorea tepejilote* and *Solanum americanum*).

For *Chamaedorea tepejilote* and *Solanum americanum* management intensification was possible because of the biological feasibility of establishing these species in agroforestry systems. This made it possible to increase their availability, meet market demand throughout the year, and ensure income generation [6, 9, 13, 17]. In contrast, for *Peperomia peltilimba* and *Laetiporus giltbersonii*, management was limited to wild gathering to meet market demand, due to their biological limitations to develop outside of their natural habitat [8, 25, 26]. In the case of *Litsea glaucescens*, harvesting was conducted from tolerated individuals, providing sufficient resources without the need for more intensive practices [12, 25]. Somewhat contrary to the observations of Ruiz-Pérez et al. [6] and Belcher et al. [5] who propose that when the market is sufficiently attractive, the product sufficiently valuable, and land/resource tenure secure, people drive a process of intensified production and household specialization among forest dwellers, resulting in increased incomes. These results show that management intensification in response to commercial demand represents only one of many factors and scenarios influencing what may occur, and that high commercial returns may also be associated with incipient management practices.

The five species mentioned in the previous paragraph make significant commercial contributions and share characteristics in common that may be relevant to their success. These include relatively high demand in terms of commercial quantities, year-round availability, and high values of cultural importance. It is generally accepted that culturally valued resources tend to have also high market demand [10, 60]. In addition, if these resources are available throughout the year, they can generate significant commercial returns for families [30]. It would be possible to expect an increase in management intensity for all five species to increase the market sales (assuming that there is sufficient demand), but the biological feasibility of manipulating these species plays an important role in the interaction between commercialization and management intensification [9, 13, 17].

The NTFPs characterized by lower commercial profitability were commercialized less frequently and in smaller quantities throughout the year, had seasonal availability, and had lower cultural importance values. These factors resulted in lower net incomes compared to the other five species, which can be commercialized throughout the year and have high cultural importance [9, 30]. This was the case for *Agave obscura* (Cacaya), *Pouteria campechiana* (Zapote amarillo), *Peperomia maculosa* (Tehuantequilitl), as well as some “quelites” and mushrooms that are only available for a few months per year, thereby limiting the possibility of consumption and commercialization.

Despite their lower commercial contribution, the intensity of management also varied between intensive, intermediate, and incipient. For some species as *Amaranthus hybridus*, *Porophyllum ruderale*, and *Canna indica*, management was intensive and focused on ensuring resource availability throw propagation and protection practices during the few months they were available. In other species, practices were simpler and relied on wild gathering as in *Arisaema macrospathum* or *Pinus* sp., and tolerance in anthropogenic environments as in *Pouteria sapota* or *Diospyros nigra,* because it is difficult to increase resource production outside their wild habitat. Typically, one of the reasons for managing and commercializing species with limited availability lies in the additional income they can provide to families [10, 60].

In Ixtacxochitla, the management and commercialization strategies are based on the use of a variety of species with specific ecological, cultural, and economic characteristics that prove beneficial to small farmers under certain circumstances or at certain times of the year. To ensure the continued presence and increased production of these resources, management practices are intensified; however, when species are difficult to manage, wild gathering and tolerance are used to meet the market demands.

Intensified management of species under continuous commercial pressure poses risks to their population persistence, particularly when wild harvesting is the primary method of meeting market demand, when resource populations are limited, or when commercial demand drives over-extraction [10, 12, 27]. According to Lotero et al. [33, 59] and Shanley et al. [61], one factor that can mitigate the risk to the populations of commercialized NTFPs is the marketing capacity of rural communities; in marginalized rural areas, poor road conditions, lack of adequate transportation, distance to markets, and long travel times limit the ability to meet large-scale market demand. As a result, commercialization predominantly relies on the extraction of multiple resources on a small scale.

In Ixtacxochitla, the difficult access conditions and reduced scale of trade decrease the risk of overharvesting for most of the species that are extracted and demanded by the market. On the one hand, the difficulty of transporting large quantities of products limits the possibility of conducting extensive harvests, especially for wild species collected in forested areas that are more difficult to access. On the other hand, since the consumption of these species is primarily concentrated in nearby communities and their trade is limited to the Coyomeapan market [33], extensive harvesting is not necessary to meet the market demand.

The management patterns of commercial species in the traditional markets of the Sierra Negra region [8, 10, 13, 34] are similar to those found in Ixtacxochitla. The extensive information provided by the interviewees, both those involved in the NTFPs trade and those who are not, allowed a deeper exploration of management strategies and the impact of factors such as frequency of use, extraction quantity, resource abundance, and income. It is important to continue conducting studies in communities where people involved in managing resources for market supply live, such as Ixtacxochitla, and it is possible to increase the number of interviewees.

## Conclusions

This research shows that the intensification of NTFPs management is not solely driven by the commercial value of the products, or the level of the net income generated. Instead, the implementation of more intensive management practices for commercialized NTFPs was driven by key factors that included the perception of lower resource abundance in the species’ natural habitat, a consistent demand for the resource throughout the year, the high cultural importance of the species, and the biological feasibility of manipulating the resource in ex situ environments. The main limitation to implementing more intensive management practices was the lack of feasibility of managing species outside their natural habitats, despite their cultural importance and frequent consumption. Factors contributing to high commercial revenues included temporal availability, consistent demand throughout the year, and the cultural importance of the species.

Analysis of the social, economic, and ecological dimensions involved in the harvesting of NTFPs provides a clearer context for management and commercialization processes, which can be helpful in understanding a presumed history of techniques and practices implemented over time in changing contexts.

Importantly, it is recognized that the social, economic, and ecological dimensions can be the basis for future research aimed at analyzing the conditions that facilitate successful and sustainable NTFPs commercialization. This, in turn, would strengthen the economies of rural families while ensuring the continued existence of the harvested species.

## Data Availability

The datasets analyzed during the current study are stored in the Base de Datos Etnobotánicos de Plantas Mexicanas (BADEPLAM) database repository at the Botanical Garden of the Institute of Biology, UNAM, and are available from the corresponding author upon reasonable request.

## Appendix 1

**Table:** List of species commercialized at the Coyomeapan market reported by the traders of the community of Ixtacxochitla

Species	Comun name		Traders in the locality of Ixtacxochitla									Total
	Nahuatl	Spanish	1	2	3	4	5	6	7	8	9	
*Solanum Americanum*	Tomaquilitl	Hierba mora	1	1	1	1	1	1	1	1	1	9
*Laetiporus gilbertsonii*	Chilnanacatl	Hongo rojo	1	1	1	1	1	1		1	1	8
*Pleurotus sp.*	Iztananacatl	Hongo blanco	1	1	1	1	1	1		1	1	8
*Peperomia peltilimba*	Tequelite		1	1		1	1	1		1	1	7
*Chamaedorea tepejilote*	Tepejilote		1	1		1		1	1	1	1	7
*Cestrum nocturnum*	Zopequilitl		1	1	1	1				1	1	6
*Auricularia delicata*	Tonagaz	Hongo oreja	1	1		1	1			1	1	6
*Schizophyllum commune*	Cashpaltzin		1	1	1	1		1			1	6
*Spathiphyllum cochlearispathum*	Ixtacxochitl	Flor blanca	1		1	1				1	1	5
*Leucaena diversifolia*	Bashi/Polonsoco	Guaje	1	1						1	1	4
*EtnoB*	Alactzin		1				1	1		1		4
*Litsea glaucescens*	Sogogotl	Laurel	1					1			1	3
*Porophyllum ruderale*	Papalo quílitl	Pápalo	1	1							1	3
*Pinus sp.*	Ocote		1						1		1	3
*Calyptranthes megistophylla*	Mototetl	Pimientón	1					1			1	3
*Witheringia solanácea*	Xaltojto		1	1							1	3
*Persea schiedeana*	Chinene	Aguacate	1								1	2
*Taluma Mexicana*	Yoloxóchitl	Flor corazón	1								1	2
*Diospyros nigra*	Tlilzápotl	Zapote negro	1				1					2
*Peperomia maculosa*	Tehuantetlquílitl		1								1	2
*Tagetes erecta*	Cempaxochitl	Flor de muerto	1								1	2
*Agave obscura*	Copalmatzin	Cacaya	1							1		2
*Pouteria sapota*	Quazapotl	Mamey									1	1
*Brassica rapa*	Colesh			1								1
*Stanophea tigrine*	Tehuaxochitl	Flor de tigre						1				1
*Peperomia sp.*	Befixivitl		1									1
*Inga sp.*	Xonohuiztli		1									1
*Amaranthus hybridus*	Bahquilitl	Quelite	1									1
*Chenopodium ambrosioides*	Apazotl	Epazote							1			1
*Renealmia Alpinia*	Veligmolli		1									1
*Arisaema macrospathum*	Neshigolispactli		1									1
*Cedrela oaxacensis*	Tiokohuitl	Cedro	1									1
*Begonia caroliniifolia*	Cuaujtómatl		1									1
*Canna indica*	Panesbatl		1									1
*Pouteria campechiana*	Holonzapotl	Zapote amarillo	1									1
*EtnoA*	Tonalxivitl		1									1
Total			32	12	6	9	7	10	4	11	20	

## Appendix 2

**Table:** Socio-ecological variables examined for the 28 NTFPs used for subsistence

Familia	Specie	Nahuatl name	Spanish name	Use	frec. con	man.int	man. fea	cul. imp	harv. sys	per. abu	Months	Mentions
Acanthaceae	*Odontonema cuspidatum*	Quachia		M	A	T	F	0.2	H,A	A	AMY	2
Actiniadiaceae	*Saurauia scabrida*	Pipitzo		E	W	G,T	F	0.2	F,R	S	A,S,O	3
Adoxaceae	*Sambucus nigra*	Xómet		M	W	T	F	0.2	R	A	AMY	3
Amanitaceae	*Amanita rubescens s.l*	Xochitecontsi		E	B	G	WM	0.2	R,A	S	Jn,Jl	4
Araceae	*Spathiphyllum wallisii*	Ixtacxochitl	Flor blanca	O	A	T,P,Pr	F	0.3	F,H	R	Ma,Jn	9
Arecaceae	*Brahea sp.*	Zoitl		O	A	G	WM	0.2	F,H	R	AMY	1
Asteraceae	*Barkleyanthus salicifolius*	Yoyotli		M	M	T,P,Pr	F	0.3	R,H	A	AMY	6
Asteraceae	*Stevia suaveolens*	Xogobashi		M		T	F	0.2	A		AMY	3
Asteraceae	*Tagetes erecta*	Cempaxochitl/ Palmatzi	Flor de muerto	C	A	T	F	0.2	R	S	S,O,N	1
Asteraceae	*Verbesina crocata*	Capitaneja	Árnica	M	A	T,Pr	F	0.2	R,H	R	A,Ma,Jn	2
Asteraceae	*Verbesina turbacensis*	Zazastli		C	A	T	F	0.3	R,M,A	A	S,O,N	7
Auriculariaceae	*Auricularia auricula-judae*	Tonagaz	Hongo oreja	E	A	G	WM	0.4	F,R,M,C	A	AMY	7
Begoniaceae	*Begonia heracleifolia*	Cecigxochitl		C	A	T	F	0.3	F,R	A	Jl,A,S,O,N,D	7
Bromeliaceae	*Catopsis sessiliflora*	Xolochi		O	M	G	WM	0.2	F,R	S	Ma, Jn,Jl,A	2
Bromeliaceae	*Tillandsia leiboldiana*	Xolochi		O	A	G	WM	0.2	F	A	Ma, Jn,Jl,A	2
Cactaceae	*Epiphyllum ackermannii*	Papalo xochitl		O	A	G,Pr	F	0.2	F	A	A	2
Cactaceae	*Nopalea cochenillifera*	Nopal		E	W	T,Pr	NF	0.2	F,R	S	F,M,A,Ma,Jn,Jl	2
Euphorbiaceae	*Croton draco*	Neskuitl		M	A	T	F	0.2	F,R	A	AMY	2
Fabaceae	*Acacia angustissima*	Opactli	Timbre	E	A	T	F	0.2	F,R	A	AMY	2
Fabaceae	*Inga tuerckheimii*	Topetli		E	A	T,Pr	F	0.2	F,R	R	AMY	2
Iridaceae	*Crocosmia aurea*		Palma cimarron	O	W	T	F	0.2	R,H	R	M,A,Ma,Jn,Jl	4
Lamiaceae	*Ocimum micranthum*	Clavoxivitl/ Ixcalxivitl		M	A	T	F	0.2	R,H	S	Jn,Jl,A,S,O	3
Lamiaceae	*Salvia purpurea*	Chíchig		M	A	T,Pr	F	0.2	R,A	A	AMY	2
Orchidaceae	*Sobralia macrantha*	Tzinagaxochitl		O	A	G,Pr	F	0.4	F	A	A,M,Jn,Jl	10
Rosaceae	*Rubus eriocarpus*	Xoxonte		M,C	B	T	F	0.3	R,C,A	A	A,Ma	3
Solanaceae	*Brugmansia arborea*	Xochitltecomatl		M	A	T	F	0.3	R,H	R	AMY	5
Solanaceae	*Lycianthes geminiflora*	Quaquilitl		E	A	G	WM	0.2	M,A	S	AMY	2
Verbenaceae	*Verbena carolina*	Chichipactli		M	A	T	F	0.2	R	A	A,Ma,Jn,Jl,A	3

Use: O, Ornamental; E, Edible; M, Medicinal; C, Ceremonial; frec. con. (consumption frequency): A, Annual/Twice per year; M, Monthly; B, Twice per week; W, Weekly; man. int. (management intensity): G, Gathered. T, Tolerated; P, Protected; Pr, Propagated; man. fea. (management feasibility): F, Feasible; NF, Not feasible; WM, Without management; cul. imp. (cultural importance); har. sys. (harvesting system): F, Forest; R, Ruderal; A, Acahual; M, Milpa; C, Coffee plantation; H, Homegarden; per. abu. (abundance perception): A, Abundant; R, Regular; S, Scace; Months: AMY, All months of the year; Ja, January; F, February; M, March; A, April; Ma, May; Jn, June; Jl, July; A, August; S, September; O, October; N, November; D, December. Mentions: number of participants mentioned the species

## Abbreviations

AP: Abundance perception
C: Comercialización
CI: Cultural importance
FC: Frequency of consumption
HS: Harvest sites
MEXU: National Herbarium of Mexico
MF: Management feasibility
MI: Management intensity
MXN: Mexican pesos
NMDS: Non-metric multidimensional scaling multivariate analysis
NTFPs: Non-timber forest products
PCA: Principal component analysis
RI: Relative cultural importance
S: Subsistence
UNAM: Universidad Nacional Autónoma de México
U: Use
Ui: Annual net income
USD: United States dollar
